# Formation of Visible Aggregates between Rolling Circle Amplification Products and Magnetic Nanoparticles as a Strategy for Point-of-Care Diagnostics

**DOI:** 10.1021/acsomega.1c05047

**Published:** 2021-11-23

**Authors:** Darío Sánchez Martín, Reinier Oropesa-Nuñez, Teresa Zardán Gómez de la Torre

**Affiliations:** † Department of Material Sciences and Engineering, Division of Nanotechnology and Functional Materials, Ångström Laboratory, Uppsala University, 751 21 Uppsala, Sweden; ‡ Department of Material Sciences and Engineering, Division of Solid-State Physics, Ångström Laboratory, Uppsala University, 751 21 Uppsala, Sweden

## Abstract

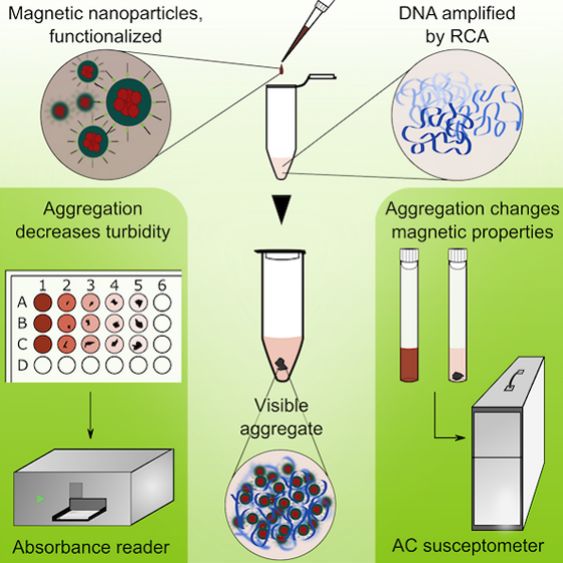

Visual detection of rolling circle amplification products (RCPs) has been achieved by specific aggregation with magnetic nanoparticles. The method presented here reliably generates aggregates in 1.5 h; these are visible to the naked eye in samples containing at least 0.4 fmol of RCPs. In addition, alternate current susceptometry and absorbance spectroscopy have also been used to quantify the amplified products. The specificity of the detection method was tested, and no non-specific aggregation was detected in samples containing up to 20 fmol of non-complementary amplified DNA. This method is a versatile tool for detecting pathogenic DNA in point-of-care diagnostics, with no readout equipment required. However, chips and automated assays can be used in conjugation with the developed method since detection and quantification can be achieved by commercially available readout instruments.

## Introduction

There is an increasing need for point-of-care (POC) pathogen diagnostics in low-income countries and at small healthcare centers. Fast methods that do not need expensive equipment, complicated machinery, or trained personnel are valuable assets in the fight against disease across the globe.^1^ These methods rely on detection of biological molecules.^2,3^

Methods that detect specific DNA sequences enable the development of a wide range of biomedical applications since all living organisms have DNA. Furthermore, these methods can usually be readily adapted to detect specific sequences of interest.^4,5^ The most common method of determining the presence of a specific DNA sequence in a sample is polymerase chain reaction (PCR) amplification, followed by confirmation of DNA presence through gel electrophoresis or fluorescence-based methods.^6,7^ However, PCR requires trained personnel and expensive materials,^6^ thus making it unsuitable for POC diagnostics. A number of alternative DNA amplification methods that are simple and efficient have been developed in recent years. One of them is rolling circle amplification (RCA), an isothermal amplification method where a DNA polymerase creates a long single-stranded DNA (ssDNA) molecule out of a circular DNA molecule.^8,9^ The circular template can be obtained using a so-called padlock probe, a linear DNA oligonucleotide phosphorylated on the 5′ end which hybridizes through both ends to a target DNA sequence. When this hybridization occurs, both ends are brought together, and a ligase can use the 5′ phosphate to link the ends, creating a circular template.^10,11^ Ligation is followed by an RCA reaction, which creates long ssDNA products of hundreds of repeats complementary to the padlock probe.^9^ The RCA products (RCPs) then collapse in random-coiled, micrometer-sized structures.^12^ RCA is suitable for POC diagnostics since it is conducted at a single temperature and does not require highly trained personnel or expensive equipment.

RCPs can be detected and analyzed using a variety of readout techniques including gel electrophoresis,^13,14^ electrochemical techniques,^15−17^ and by colorimetric^18−20^ and spectroscopic visualization.^21,22^ One of the most common techniques to detect RCPs is optically, using fluorescence analysis.^23^ This readout method is based on the binding of agents to the amplified DNA, giving a fluorescent signal, or through fluorescence-labeled detection oligonucleotides (DOs) that are complementary to the repeating sequence in the RCPs. Because fluorophores are light sensitive and need to be handled and stored properly, they can be unsuitable for POC diagnostics. As an alternative, magnetic nanoparticles (MNPs) can be used as labels where their signal can be measured by magnetometers. MNPs are physically stable, easy to handle, and not sensitive to light.^24^ To date, many different types of sensors that can measure the magnetic field from magnetic particles have been developed.^25,26^ Among magnetic field sensors commonly used for biosensing are superconducting quantum interference devices (SQUIDs),^27−29^ inductive methods,^30−32^ fluxgates,^33−35^ and magnetoresistive sensors.^36,37^ The SQUIDs are very sensitive, but they are large in size and require cryogenics, which makes them expensive and not suited for POC. Sensors based on induction, fluxgates, and magnetoresistivity are smaller in size and more flexible in their design. Due to their smaller size, they often require smaller sample volumes and can potentially be integrated with a microfluidic sample preparation device.

The Brownian relaxation detection principle is an inductive method where MNPs are coupled to DOs that have the ability to bind specifically to complementary RCPs. The Brownian relaxation frequency of the MNPs changes to lower frequencies when they are bound to RCPs as these molecules are much bigger in size than the MNPs. The change in the relaxation frequency can be measured by alternate current (AC) susceptometry. This is the underlying working principle of the volume-amplified magnetic nanobead detection assay (VAM-NDA).^38,39^ Tabletop AC susceptometers are commercially available and have been used for VAM-NDA. An earlier study has shown that RCPs can be detected in around 2 min in a portable AC susceptometer.^40^ Recent research has also demonstrated that different types of nanoparticles can form aggregates in the presence of the analyte of interest, leading to visual identification of the reaction products.^22,41,42^ This has been carried out in combination with PCR,^41^ or with RCA, using gold^22^ or MNPs.^42^ However, the studies using RCA indicated practical limitations such as long assay times (>3.5 h), the need for in-lab synthesis of nanoparticles, and non-specific binding of the particles, which can easily lead to false results.

In this work, we explored the possibility of combining the VAM-NDA with MNP aggregation to develop a biosensing method for POC diagnostics based on visual detection. Synthetic *Vibrio cholerae* DNA was used as a model target sequence for this study.

## Results and Discussion

We have developed a detection method based on the formation of visible aggregates resulting from the interaction of MNPs and RCPs. The colloidal particles in the MNP solution used in the study gave the solution a dark brown color. The hue of the solution became lighter when the MNPs and RCPs aggregated and precipitated. This allowed a multi-readout assay using naked eye detection of precipitated MNP-RCP aggregates, absorbance/turbidity measurement of the solution color, and AC susceptibility readout.

**Figure 1:**
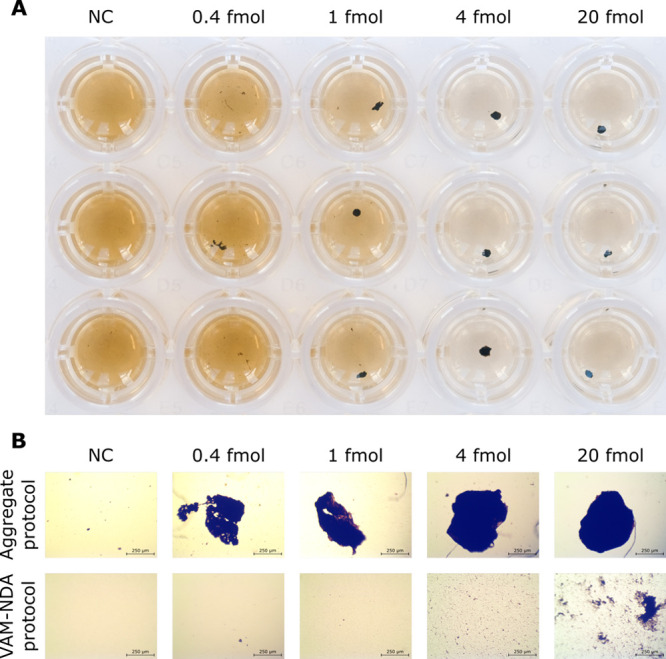
Aggregates can be seen with the naked eye and under a microscope. (A) 96-well plate containing aggregates formed in samples with different RCP amounts using the aggregation protocol. (B) Comparison of samples using the aggregate and VAM-NDA protocol under an optical microscope (4× objective). Photographs were taken by T. Zardán Gómez de la Torre and micrographs were taken by D. Sańchez Martín.

**Scheme 1:**
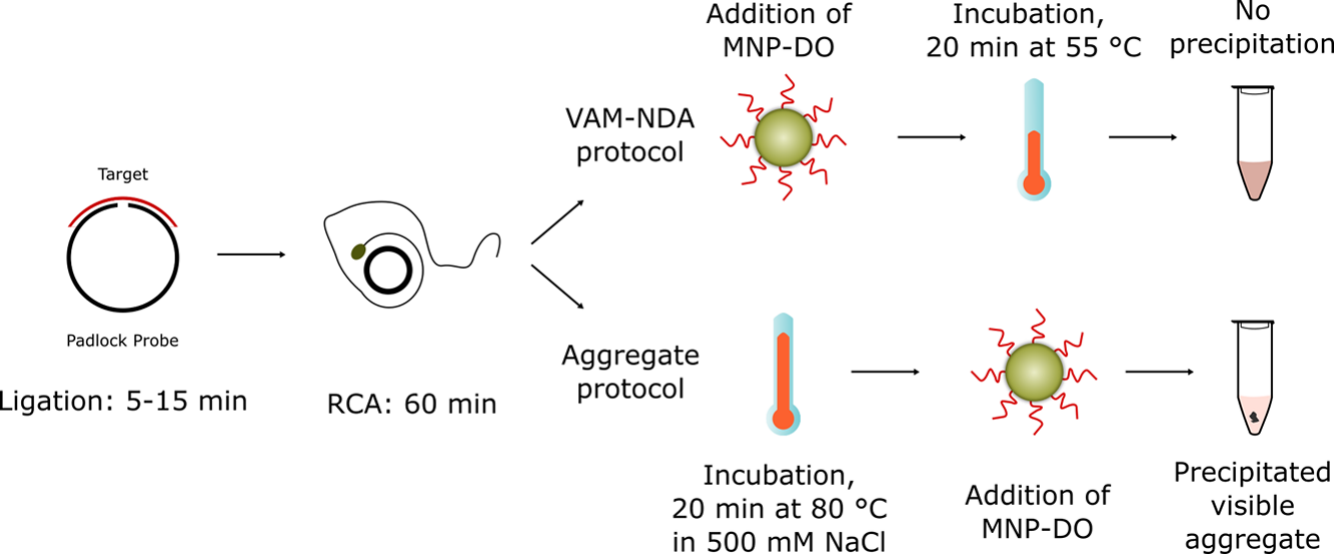
Comparison of the Two Methods; Schematic Showing the Differences between the VAM-NDA and the Aggregation Protocol DO = detection oligonucleotides; MNP = magnetic nanoparticles; RCA = rolling circle amplification; and VAM-NDA = volume-amplified magnetic nanobead detection assay.

This approach yields results in around 1.5 h with samples containing as low as 0.4 fmol of RCPs (in theory each target molecule yields a maximum of an RCP). We optimized the incubation step of the VAM-NDA so that the MNPs could bind to the RCPs to form millimeter-sized aggregates that easily precipitated to the bottom of the sample tubes. The differences between the two protocols are outlined in Scheme 1. Figure 1A shows how the formed aggregates are visible to the naked eye, facilitating detection of specific DNA targets, as no readout system is required. As can be seen in Figure 1B, the aggregation protocol resulted in compact, individual aggregates even with the lowest RCP amounts (0.4 fmol). In contrast, the VAM-NDA protocol generated more disperse and smaller clusters and only in samples between 4 and 20 fmol of RCPs.

MNPs and RCPs cannot bind and aggregate at room temperature (Figure S1). Incubation at a high temperature is required to open the collapsed RCPs and allow MNP binding. Aggregation was also enhanced by high NaCl concentration. NaCl concentrations of 1 M or less raises the melting temperature of double-stranded DNA,^44^ allowing stronger binding of the DOs, as it balances the negative charges of DNA. Several NaCl concentrations and temperatures were analyzed for the incubation step to form the aggregates (Figure S2). It was found that using 500 mM of NaCl and incubation at 80 °C were the optimal conditions to form compact aggregates. We presume that the high temperature and high NaCl concentration provide a good environment for the RCPs to open and the MNPs to bind. This results in RCPs and MNPs forming loose viscous aggregates in less than 2 min. Figure S3 contains images showing the process of aggregation when the MNPs were mixed with the RCPs. Once the tubes were spun down or the samples left to cool, the aggregates collapsed and precipitated, becoming tight and stable, as shown in Figure 1A. Heating or vortexing did not separate the aggregates, although vortexing caused them to break into smaller pieces. Others have reported that non-functionalized gold NPs aggregate at room temperature in the presence of salt.^45^ This was not the case for our MNPs as can be seen by the lack of aggregation in the negative control (NC) samples. We used atomic force microscopy, specifically magnetic force microscopy, to ensure that the aggregates contained both DNA and MNPs. Images are shown in Figure S4. We observed MNPs of the expected size, along with what seemed to be ssDNA.

**Figure 2:**
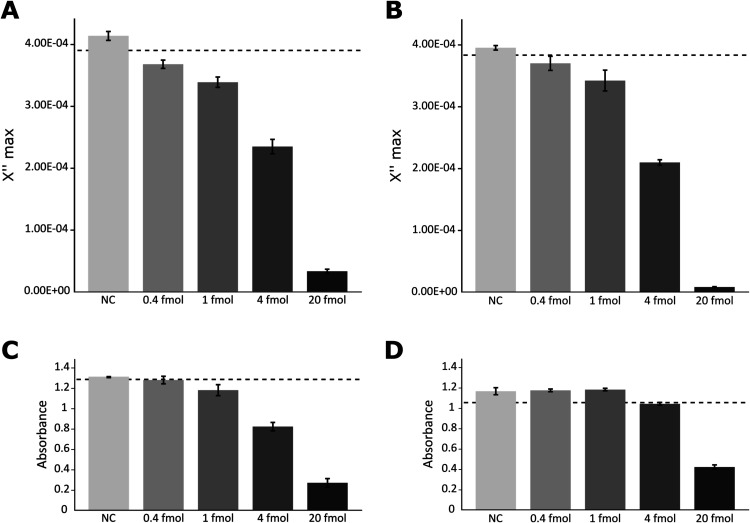
Number of RCPs can be quantified using an AC susceptometer and an absorbance reader. (A,B) Average peak maximum of the imaginary part of the complex magnetization spectra (at 128 Hz) for the aggregate (A) and VAM-NDA (B) protocol samples. (C,D) Absorbance values at 350 nm of samples using the aggregate (C) and VAM-NDA (D) protocols. Error bars represent standard deviation (sd) based on *N* = 3. The dashed line indicates the values for the NCs minus 3*sd, representing the LOD.

Visual identification of the aggregates does not provide a quantitative estimate of DNA in the sample. In the VAM-NDA approach, immobilized MNPs have a lower Brownian relaxation frequency than free MNPs, as detected in a commercially available tabletop AC susceptometer. We assumed that the same was true for the aggregate approach. The magnetization responses for both detection methods are presented in Figure 2A,B, and the magnetization spectra are presented in Figure S5. At 0.4 fmol of RCPs, both approaches resulted in a significant drop in the values of the imaginary part of the frequency peaks, which represent unbound MNPs with a nominal size of 100 nm. The peaks decreased with higher amounts of RCPs, becoming nearly flat at 20 fmol. There was no change in the limit of detection (LOD) (as defined in Materials and Methods) for the new method compared to the VAM-NDA, meaning that accurate quantification of RCPs is still possible with the aggregate approach.

**Figure 3:**
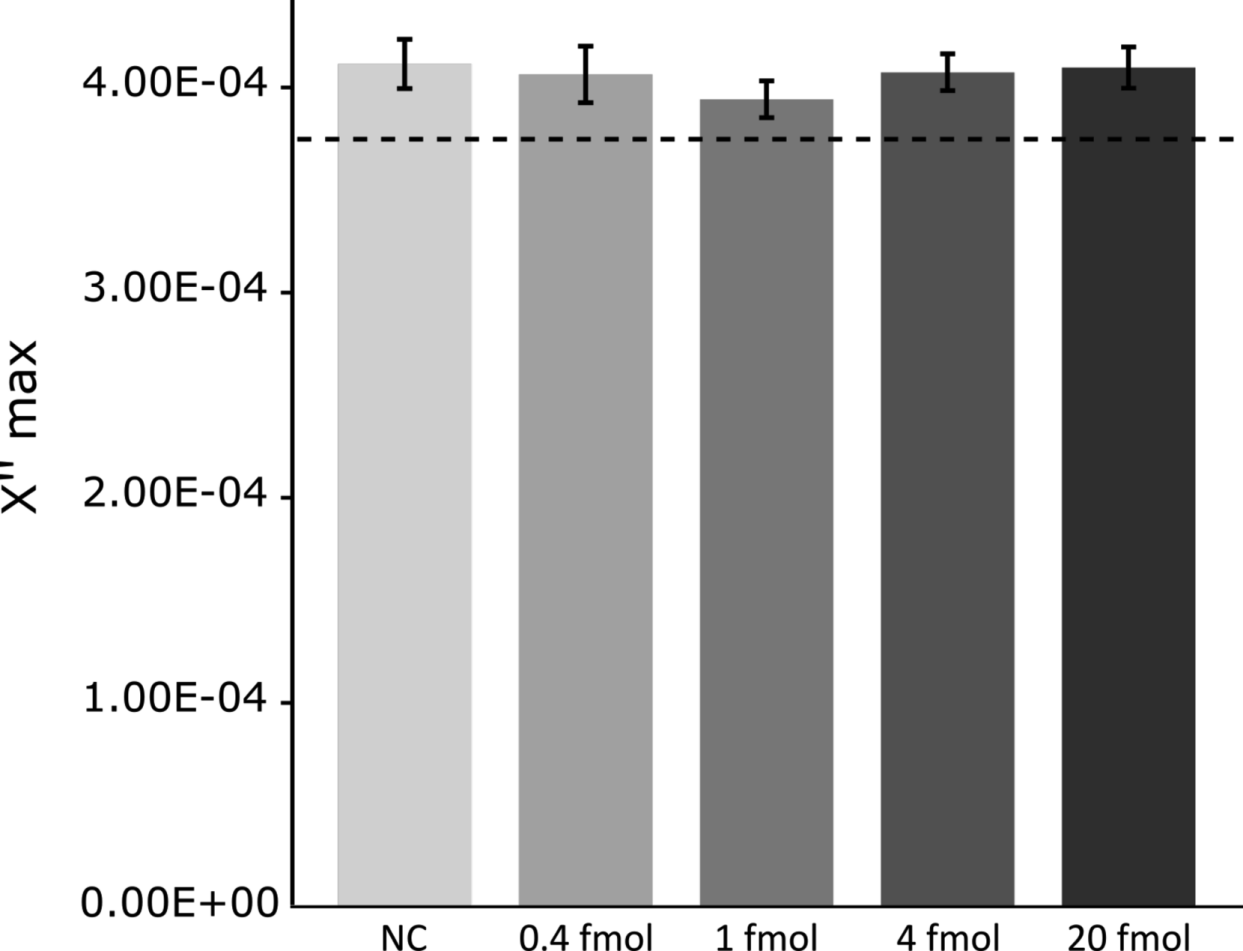
Aggregation of MNPs is specific to DNA that is complementary to the DOs. Average peak maximum of the imaginary part of the complex magnetization spectra (128 Hz) for different RCP amounts and MNPs with non-complementary DOs. Error bars represent sd based on *N* = 3. The dashed line indicates the values for NCs minus 3*sd, representing the LOD.

The formation of aggregates makes the sample solutions clearer than the solutions in the NC samples (see Figure 1A). This is due to the large aggregates precipitating out of the solution. We theorized that an absorbance measurement at a short wavelength, outside the absorption range of DNA molecules, could be used to quantify unprecipitated MNPs in the sample. The results are shown in Figure 2C,D. With the aggregate protocol, there was a significant difference from the NCs in samples containing 1, 4, and 20 fmol of RCPs but not in samples containing 0.4 fmol. This was not the case with the VAM-NDA protocol, where there was a substantial difference from the NCs only in samples containing 20 fmol of RCPs. There was only a small difference from the NCs for the 4 fmol samples and no difference for the samples containing 0.4 and 1 fmol. Thus, the aggregation protocol allows better quantification through absorbance than the VAM-NDA method. This is an advantage of the new approach since absorbance spectroscopy is a simple readout technique. In addition, several samples can be placed on a 96-well plate and measured at the same time in a matter of minutes, making the protocol faster and allowing automatization. The possibility of using an absorbance reader makes the method much more useful as many laboratories and health centers around the world have absorbance readers nowadays. Lastly, we addressed the specificity of the new approach. The padlock probe-based RCA protocol has good specificity because the padlock probe is ligated only when a complementary sequence is present.^10^ However, it is possible that DNA contaminants are present in a sample or even that unwanted circularization and amplification of the DNA target might occur. To test the specificity of the aggregate formation, we changed the DO bound to the MNPs to a DO with a sequence that was not complementary to the RCPs. No aggregation was observed, and the AC susceptometry results confirmed the absence of aggregates. The results are shown in Figure 3; there were no significant differences between samples ranging from 0.4 to 20 fmol of RCPs and the NCs. These results indicate that the aggregation protocol is specific. The protocol is thus reliable as it depends on the specificity of both the padlock probe ligation and the DO recognition of RCPs. Other research has been carried out where MNPs show to aggregate with RCPs independently of their sequence,^42^ but that is not the case here.

Table 1 presents several DNA detection methods based on isothermal amplification and optical detection. The sensitivity of our method varies from 0.4 to 1 fmol (2–5 pM in 200 μL) depending on the readout technique. Our detection method is more sensitive than the detection techniques based on nucleic acid sequenced-based amplification (NASBA) and hybridization chain reaction (HCR). Our method has also the advantage to be more rapid compared to these detection methods. The assays based on RCA have as good as or better sensitivity compared to our method but lack in detection speed. These assays have also showed other practical limitations, such as in-lab synthesis of the nanoparticles, and non-specific binding of the particles to the RCPs, which has not been observed in the current study.

**Table 1:** Comparison of Different DNA Detection Methods Based on Isothermal Amplification and Optical Detection

amplification method	readout technique	LOD	assay time	reference
NASBA	fluorescens	0.1 μM	about 2 h	(46)
HCR	colorimetric (AuNP)	500 pM	about 4 h	(47)
HRCA	colorimetric (phenol red)	3 pM	about 3 h	(18)
RCA	colorimetric (AuNPs)	70 fM	less than 4 h	(22)
RCA	visual (magnetic particles)	0.62 fmol	6 h	(42)
RCA	visual, spectroscopy, and magnetic	0.4–1 fmol (2–5 pM) depending on the readout	less than 2 h	this work

Overall, using RCA with functionalized MNPs provides same or in some cases an increase in sensitivity but offers a more rapid detection speed compared to most of the presented methods. This makes it a robust method for POC diagnostics in healthcare centers lacking experienced laboratory personnel or laboratories with sterile conditions.

## Conclusions

We have demonstrated that this detection approach creates visible aggregates from a DNA target sequence amplified through RCA and commercially available MNPs. The method provides high sensitivity and specificity coupled with visual and instrumental readouts, and the entire assay used here takes less than 2 h. It not only keeps the sensitivity of the VAM-NDA in a tabletop AC susceptometer, such as the DynoMag, but also allows visual identification and optical quantification through absorbance measurements. The most important factor is the potential for running samples in a 96-well plate, which would allow fast quantification of many samples at the same time. Visual identification without readout equipment is also possible if a qualitative response (positive/negative result) is all that is needed. In addition, the protocol is shown to be very specific.

The proposed method requires long ssDNA products with complementary sequences to a DO to form visible aggregates, and while we based our method in the VAM-NDA, aggregation of MNPs to RCPs is likely to happen if we were to use other RCA-based techniques producing ssDNA (such as circle-to-circle amplification) or perhaps even loop-mediated isothermal amplification.

The purpose of the study was to showcase the aggregation step and how that opens avenues into naked-eye detection or absorbance-based quantification for POC diagnostics. Nevertheless, many questions remain about the aggregation itself. It has shown to be highly specific, and also to require high temperature and salt concentration, and we intend to investigate this phenomenon further to improve the aggregation of RCPs and MNPs when it comes to sensitivity, time, and ease of use. We hope this novel method will aid in the design of robust, specific, and sensitive diagnostic tests for POC diagnostics.

## Materials and Methods

### MNPs and Reagents

The core–shell MNPs used in this study were purchased from Micromod Partikeltechnologie, Germany. The MNPs had a core of 75–80 wt % magnetite, which was encapsulated in cross-linked hydroxyethyl starch, with surface streptavidin (product code 10-19-102). These particles had a diameter of around 100 nm and were suspended in phosphate-buffered saline (PBS) at a concentration of 10 mg/mL. The theory behind the dynamic magnetic properties of the NPs is outlined in supporting theory in the Supporting Information.

All oligonucleotides were purchased from Biomers, Ulm, Germany, and all reagents were purchased from Thermo Fisher Scientific, Waltham, MA, USA, unless otherwise specified. The oligonucleotides used in this work are listed in Table 2.

**Table 2:** Oligonucleotides Used in This Work

name	sequence
padlock probe *V. cholerae*	5′-taggttgagcccagggacttctagagtgtaccgacctcagtagccgtgactatcg acttgttgatgtcatgtgtcgcaccaaatgcgattcc-3′
target *V. cholerae*	5′-ccctgggctcaacctaggaatcgcatttg-3′
DO *V. cholerae*	biotin-5′-ttttttttttttttttttttgttgatgtcatgtgtcgcac-3′
DO C2CA *V. cholerae*	biotin-5′-ttttttttttttttttttttgtgcgacacatgacatcaac 3′

### Ligation and RCA Reaction on Synthetic Target DNA

A ligation reaction to circularize 20 nM of padlock probes was performed (1× phi29 buffer, 1 mM ATP, 20 nM padlock probe, 60 nM *V. cholerae* synthetic target DNA, 0.1 U/μL T4 ligase) for 15 min at 37 °C. The circularized padlock probes were then amplified in an RCA reaction (6.66 nM circularized padlock probe, 1× phi29 buffer, 0.2 mg/mL bovine serum albumin, 0.25 mM dNTPs, and 0.33 U/μL phi29 polymerase) for 1 h at 37 °C followed by a 5 min enzymatic inactivation at 65 °C.

### Functionalization of MNPs with DO and Hybridization between Functionalized MNPs and RCPs

#### Aggregation Protocol

Non-functionalized MNPs were washed three times with washing buffer [6.6 mM Tris-HCL pH 8, 3.3 mM ethylenediaminetetraacetic acid (EDTA), 66 mM NaCl, 0.06% Tween-20] using a permanent magnet. The MNPs were then resuspended in washing buffer with 240 nM of DO *V. cholerae* and an MNP concentration of 4 mg/mL, and the mixture was incubated for 1 min at room temperature.

The formation of visible aggregates was achieved as follows: the RCPs were diluted to the desired concentration with a 500 mM NaCl solution. 20 μL of the RCA solution was incubated in a thermoblock at 80 °C for 20 min, and 5 μL of the functionalized MNPs (4 mg/mL) was added immediately after. The tube was lightly vortexed. The aggregates were formed at room temperature over about 2 min, and the solution was then spun down to collect condensed water at the bottom of the tube.

For the optimization of the aggregate protocol, the following NaCl concentrations and incubation temperatures were analyzed: 250 mM, 500 mM, 750 mM, and 1 M NaCl and 60, 70, 80, and 90 °C, respectively.

#### VAM-NDA Protocol

This procedure closely followed that of Oropesa-Nuñez et al*.*^43^ Briefly, after the MNPs were washed and functionalized, as described previously, they were resuspended in PBS to a concentration of 1 mg/mL MNPs and 60 nM DO *V. cholerae*. The RCPs were serially diluted with hybridization buffer (0.1 M Tris-HCl pH 8, 0.1 M EDTA, 0.5% Tween 20, 2.5 M NaCl) to achieve the desired RCP concentrations. 20 μL of RCPs was mixed with 20 μL of functionalized MNPs and incubated for 20 min at 55 °C. Measurements were performed immediately after.

### AC Susceptibility Measurements

Samples were pipetted into vials, and a final volume of 200 μL was obtained by adding PBS. The frequency-dependent magnetic susceptibility measurements were conducted in an AC susceptometer (DynoMag, RISE Acreo, Göteborg, Sweden) at room temperature from 5 to 250,000 Hz at 21 logarithmically equidistant frequency points. Triplicates were run for each sample type.

### Absorbance Measurements

Samples with DNA, NCs (no DNA), and buffer blanks (no MNPs) were placed in a 96-well plate (transparent and flat wells), and PBS was added to bring the volume to 200 μL/well. Individual absorbance measurements were carried out at 350 nm with a 10 ms settle time and 4 × 4 filled circle multi-well reads (1500 μm away from well edges) in an Infinite 200 plate reader (Tecan, Sweden). Blank values were subtracted from all samples. Triplicates were run for each sample type.

### Imaging of DNA Samples

Optical microscopy was used to evaluate the morphology of the aggregates. The samples were photographed on a 96-well plate with an Olympus BX60 optical microscope and an OMAX A3580U3 camera (Figure 1B).

The samples were also photographed with a Fujifilm X-T3 camera and a Fujinon XF 90/2 R LM WR lens (Figure 1A).

### Specificity Measurements

To test the specificity of the aggregation step, RCPs at different concentrations were incubated, as described previously. After the incubation, MNPs functionalized with DO C2CA *V. cholerae*, a sequence not complementary to the RCPs, were added to the RCPs. The samples were then assessed in an AC susceptometer, as described previously. Triplicates were run for each sample type.

### Limit of Detection

The LOD was defined as the average value of the NCs minus three times the sd. A sample was considered detectable if its value plus sd was below the LOD. The LOD was calculated for both absorbance and AC susceptibility measurements.

## Supporting Information Available

Theory behind the dynamic magnetic properties of MNPs, optimization of the aggregation protocol, images of the aggregation process, AFM/MFM micrographs of the aggregates, and raw data corresponding to the AC susceptometry measurements (PDF)
