# Supplementary material for: Formation of Visible Aggregates between Rolling Circle Amplification Products and Magnetic Nanoparticles as a Strategy for Point-of-Care Diagnostics
Source: ACS Omega. 2021 Nov 23;6(48):32970–6. doi: 10.1021/acsomega.1c05047 (PMC8655940; doi:10.1021/acsomega.1c05047)
Supplement: Supplementary file 1 — ao1c05047_si_001.pdf [file ao1c05047_si_001.pdf]

Supporting Information for

Formation of visible aggregates between rolling circle amplification products and magnetic nanoparticles as a strategy for point-of-care diagnostics

Darío Sánchez Martín[1], Reinier Oropesa-Nuñez[2,] and Teresa Zardán Gómez de la Torre[1, *]

[1]Department of Material Sciences and Engineering, Division of Nanotechnology and Functional Materials, Uppsala University, Ångström Laboratory, Uppsala, Sweden

[2]Department of Material Sciences and Engineering, Division of Solid-State Physics, Uppsala University, Ångström Laboratory, Uppsala, Sweden

Theory

Dynamic magnetic properties of magnetic nanoparticles

The magnetic particles used in this work are composed of a cluster core consisting of 2-4 nm single domain iron-oxide nanoparticles held together by a biocompatible casing. The nanoparticle magnetic moments are thermally blocked at room temperature. The Debye theory gives an expression for the complex low-field susceptibility $\chi(\omega)$ for an ensemble of particles according to

$$\chi(\omega) = \frac{\chi_0 - \chi_\infty}{1 + i\omega\tau} + \chi_\infty \qquad \text{(S1)}$$

where ω is the angular frequency of the applied AC magnetic field, χ_∞ is the high-frequency susceptibility, χ_0 is the low-field equilibrium susceptibility, and τ is the characteristic relaxation time of the beads.

The magnetic particle relaxation is governed by either of two relaxations mechanisms: The Néel relaxation, where the magnetic moment rotates within the iron-oxide single-domain nanoparticles, or the Brownian relaxation, where the single-domain nanoparticle magnetic moments are blocked and the entire particle rotates in response to the field. The prevailing relaxation mechanism for particles used in the present work is the Brownian relaxation mechanism. The characteristic relaxation time of the Brownian relaxation model is given by

$$\tau_B = \frac{3\eta V_B}{kT} \qquad \text{(S2)}$$

where V_B is the bead hydrodynamic volume, kT is the thermal energy, and η is the dynamic viscosity of the carrier liquid. The Brownian relaxation frequency, $f_B = (2\pi\tau_B)^{-1}$ is the frequency characterizing the position of the peak in the χ'' vs. frequency spectrum.

In the VAM-NDA, the binding of particles causes a decrease of the Brownian relaxation frequency, since it is inversely proportional to the hydrodynamic volume of the beads. The

number of RCPs can then be monitored by measuring the decrease of the amplitude of the Brownian relaxation peak of the remaining free particles.

Results

Incubation of RCPs and MNPs at room temperature

The aggregation protocol was used in a sample containing 4 femtomoles of RCPs but instead of incubating the sample at 80 ˚C, the sample was incubated at room temperature. After addition of the MNPs, the sample was pipetted after a few minutes into a glass slide and imaged. One can see that no aggregates are formed in the sample incubated at room temperature.

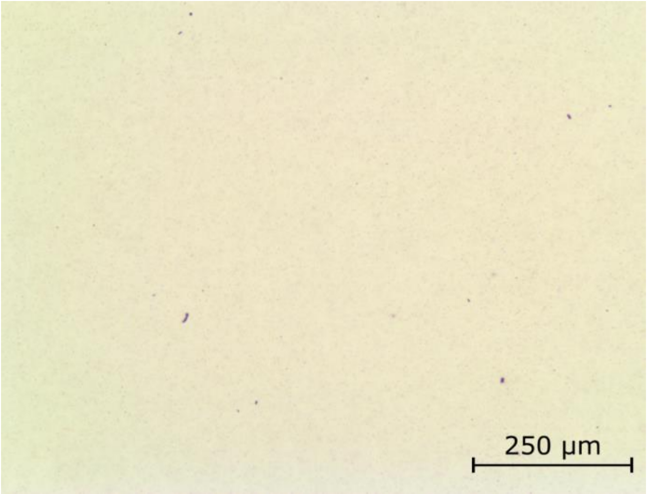

Figure S1: Aggregation does not occur at room temperature. Optical microscopy image of a sample containing 4 femtomoles of RCPs, after addition of functionalized MNPs at room temperature. No aggregates are formed.

Optimization of the aggregation protocol

The incubation of RCPs was optimized for the aggregation protocol. Different temperature and NaCl concentrations were tested in order to find the optimal conditions to form compact aggregates. Samples containing 1 femtomoles of RCPs were subjected to different NaCl concentrations (250 mM, 500 mM, 750 mM and 1M NaCl) and incubation temperatures (60 ˚C, 70 ˚C, 80 ˚C and 90 ˚C) and the results were compared to its respective negative control sample. The negative control samples contained the same amount of MNPs as the positive samples, and a 500 mM NaCl solution that was heated up to 80 ˚C.

From Figure S2 it can clearly be observed that using 500 mM NaCl at 80 ºC resulted in a higher degree of MNP binding to the RCPs creating compact aggregates.

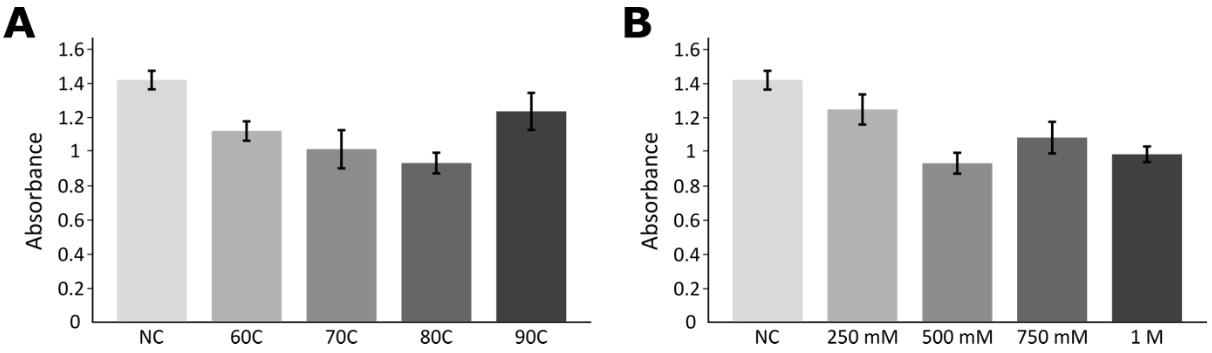

Figure S2: Optimization of the incubation step. Absorbance readouts performed to determine the optimal (**A**) incubation temperature and (**B**) salt concentration. Error bars represent standard deviation based on N=3.

Imaging the aggregation process

Samples to image the process of aggregation were prepared as follows prior imaging: the RCPs were incubated according to the aggregate protocol (see section 4.1). The MNPs were pipetted onto the tube with the heated RCPs and all contents of the tube immediately pipetted onto a transparent clear plastic under the microscope. Images were taken at room temperature.

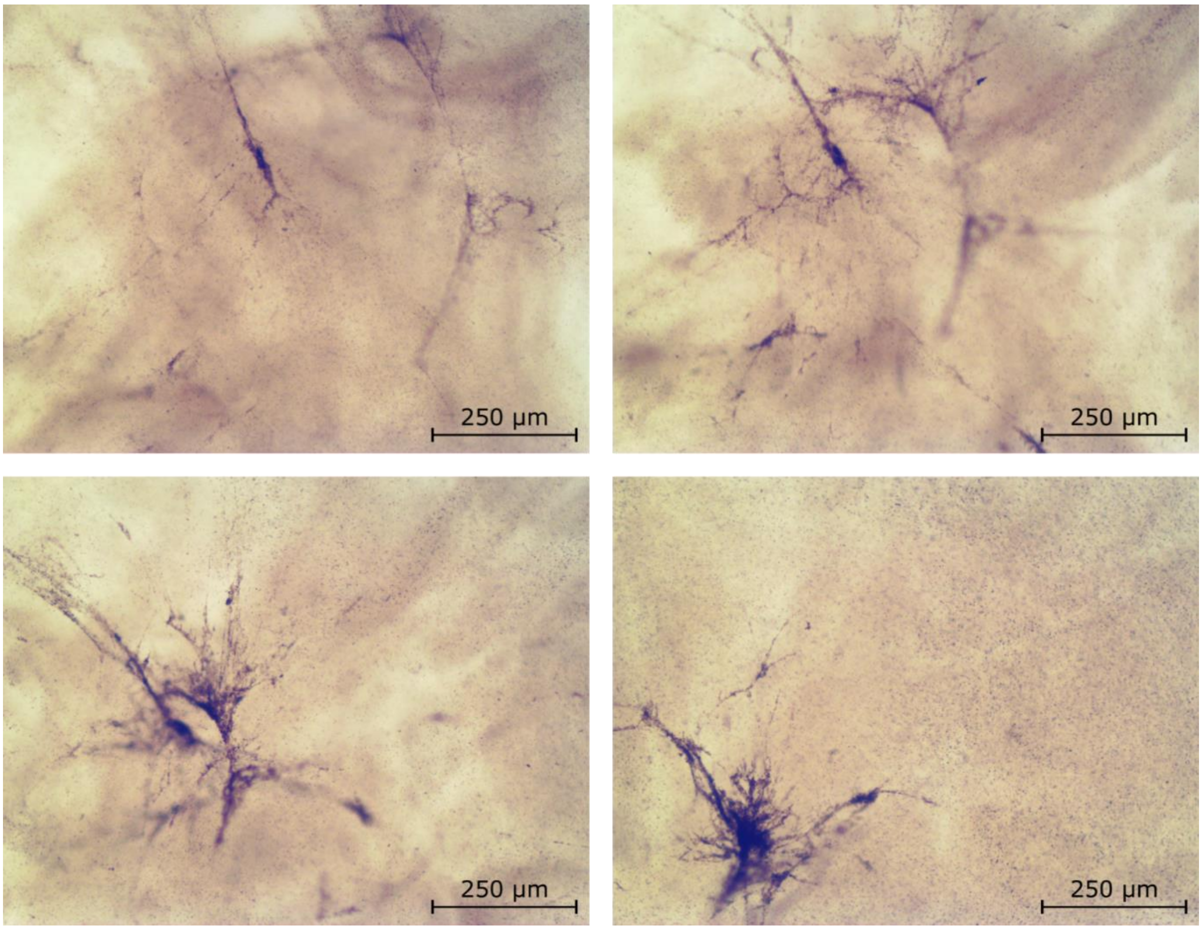

Figure S3: Formation of an aggregate under the microscope. Images of RCPs and MNPs aggregating on a plastic surface under an optical microscope. MNPs bind to RCPs and collapse quickly to form denser aggregates. Images taken of the early moments of aggregation of a sample with 4 femtomoles of RCPs.

Atomic Force Microscopy/Magnetic Force Microscopy

The aggregates were investigated using a Bruker Dimension Icon atomic force microscope (AFM) (Bruker Dimension IconTM, Billerica, MA, USA). In particular, the magnetic force microscopy (MFM) mode was used. In this mode, the morphology, and the magnetic behavior of the remnant magnetic domains in the sample surface are studied using an AFM tip coated with a Co/Cr thin film. For the experiments, a tip of typical curvature radius of 35 nm, a nominal spring constant of 3 N/m and resonance frequency in air ranging from 45 kHz to 90 kHz were used. MFM measurement was performed at a lift height of 50–100 nm. Before the MFM measurements, the AFM cantilever was magnetized. Scan areas of 3×3 μm^2 (2048 points per line) were analyzed. The AFM images were processed using the Gwyddion 2.54 software.

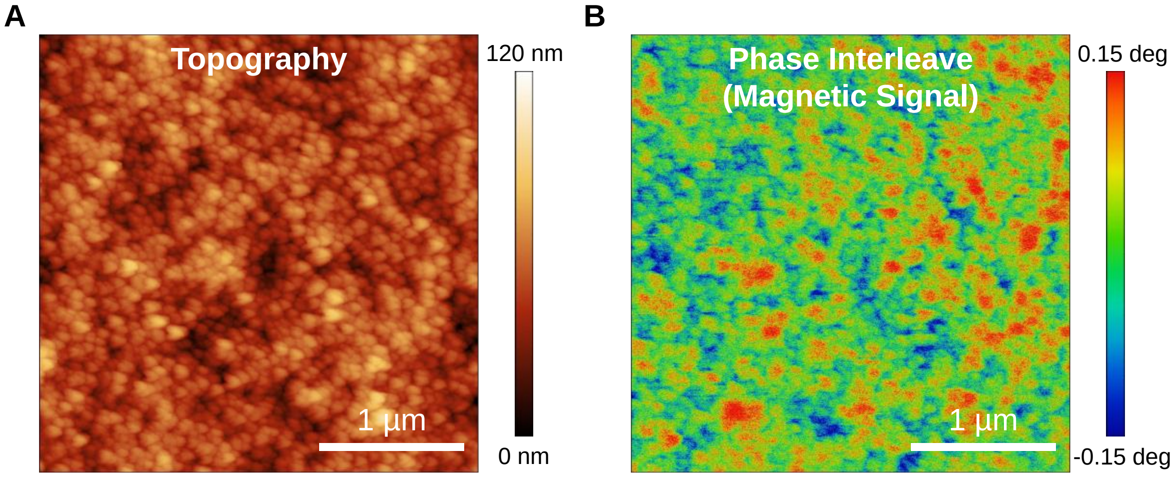

Figure S4: MFM images of DNA-MNPs aggregates. (**A**) Morphology and (**B**) the magnetic signal (MFM signal) of DNA-MNPs aggregates obtained from an aggregate in a sample containing 4 femtomoles of RCPs.

AC susceptometry measurements

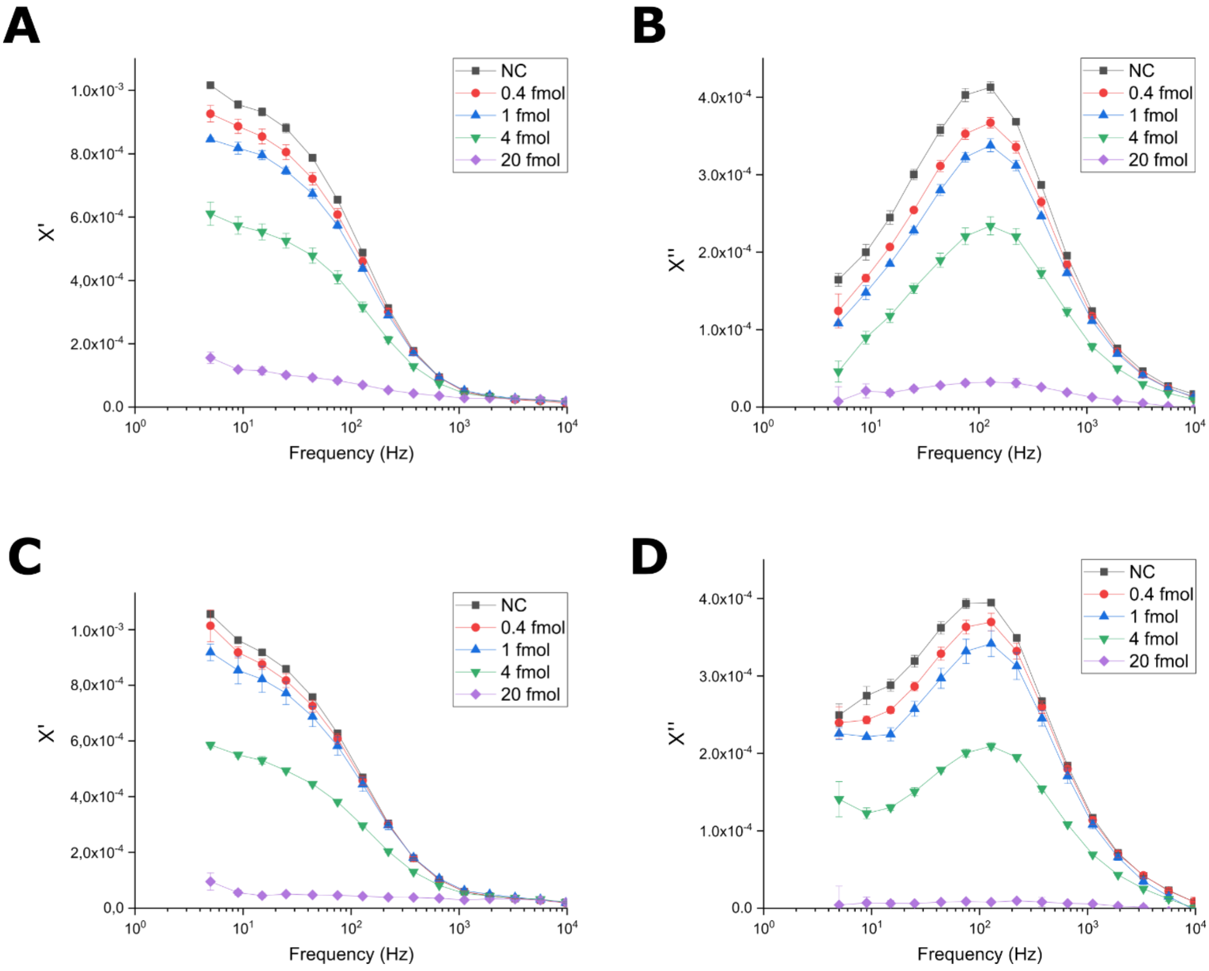

Figure S5: <u>Comparison of the protocols using AC susceptometry</u>. Real and imaginary parts of the complex susceptibility spectra for the aggregate (**A and B**) and the VAM-NDA protocol (**C and D**). Error bars represent standard deviation based on N=3.
